# Supplementary material for: A Quantum Field Approach for Advancing Optical Coherence Tomography Part I: First Order Correlations, Single Photon Interference, and Quantum Noise
Source: J Lasers Opt Photonics. Author manuscript; Available in PMC 2018 May 30. (PMC5976263; doi:10.4172/2469-410X.1000176)
Supplement: Appendix [file NIHMS963882-supplement-Appendix.docx]

**Appendix: Measurement and Decoherence**

This appendix is for those who do not routinely work in quantum mechanics. It seeks to give an overview of the difference between decoherence and measurement. In the text we discuss decoherence and measurement (the latter popularly but inaccurately often termed ‘collapse of the wavefunction’), two concepts that still are far from fully understood. In the Young’s interferometer example, measurement is occurring at the screen while decoherence is occurring from reversible interactions with the environment (E). We operate under the description of reality that is non-local and where classical space-time is a manifestation of ‘measurement’ [3]. This is a concept we have described in detail elsewhere [64].

John von Neumann’s version of measurement or ‘collapse of the wavefucntion’ has received the most attention for over the last three quarters of a century, so is presented here, where he viewed [18].

**Measurement as a transition from a quantum state into a classical state [1]:**

A quantum state:

-The deterministic, unitary, continuous time evolution of an isolated system that obeys Schrodinger’s equation (or a relativistic quantum field theory or even more recent theories such as string theory). The state is generally a superposition of classical states until measurement. [More recent work would expand this to be non-local].

Measurement:

-The probabilistic, nonunitray, discontinuous change into a specific eigenvalue. In this view the ‘observer' experiences a jump or collapse into a classical state.

In general, quantum systems exist in superpositions of basis states (eigenstates) which when not being measured or observed, evolve according to the time dependent Schrödinger equation or some equivalent evolution equation. However, when a measurement is taken, from an observer's perspective the state "leaps" or "jumps" to just one of the basis states and uniquely acquires a value of the property being measured. After the collapse, the system begins to evolve again according to the Schrödinger’s equation or some equivalent time evolution equation.

Whether there is actually a discontinuity actually exists is a subject of considerable debate. Alternative approaches to this “measurement problem” than decoherence include Everett's relative interpretation and De Broglie-Bohm theory, but current opinion favors the decoherence based approaches (none of these approaches completely addresses the measurement process) [59,60]. Irrespective, measurement is an irreversible process where the system ends up in one of several eigenvalues. What state results is not deterministic and is dictated by probabilistic rules. The results of a single measurement can’s be known a priore (with few exceptions) but the distribution of results of a large numbers of interactions can be known with extreme accuracy.

Decoherence can be thought of entanglement (reversibly or irreversibly) of the state (completely or in part) with the environment [4, 23-25]. This was illustrated with Young’s experiment in figure 1. It is purely a quantum mechanical phenomenon. If it results in an irreversible loss of path indistinguishability at measurement, there is a loss of the original coherence. We will see how decoherence in part (but not completely) deals with the measurement problem.

The measurement problem (quantum to classical transition) generally has three components [25]:

1. The problem of preferred basis. An example is why are physical systems usually observed to be in definite positions rather than in superpositions of position.
2. The problem of non-observablity of interference.
3. The problem of outcomes. Why do measurements have outcomes at all and what selects out a particular eigenvalue among the different possibilities described by the quantum probability distribution.

Decoherence seems to address 1 and 2. With respect to one, decoherence induced pointer states (environmentally selected states for examples), select out a preferred basis. With respect to two, loss of interference occurs when indistinguishable paths become distinguishable via environmental interactions (described in text). In general for decoherence to occur, the environment that the state is entangled to is different for each potential path, environmental elements do not significantly have correlations with each other, and rapidly dissipates the interaction to other environmental elements (large degree of freedom).

However, decoherence does not appear to provide an answer to the third component of measurement. Measurement results in specific eigenstates that occur with quantum mechanical probability distributions [3]. To date, a generally accepted theory how (or if) decoherence can select out specific eigenvalues with specific probabilities of occurring has yet to be accepted.
